# Humoral and cellular immune response in Wistar Han RCC rats fed two genetically modified maize MON810 varieties for 90 days (EU 7th Framework Programme project GRACE)

**DOI:** 10.1007/s00204-018-2230-z

**Published:** 2018-05-31

**Authors:** Jana Tulinská, Karine Adel-Patient, Hervé Bernard, Aurélia Líšková, Miroslava Kuricová, Silvia Ilavská, Mira Horváthová, Anton Kebis, Eva Rollerová, Júlia Babincová, Radka Aláčová, Jean-Michel Wal, Kerstin Schmidt, Jörg Schmidtke, Paul Schmidt, Christian Kohl, Ralf Wilhelm, Joachim Schiemann, Pablo Steinberg

**Affiliations:** 1 Faculty of Medicine, Slovak Medical University, Limbová 12, 83303 Bratislava, Slovakia; 2 Service de Pharmacologie et d’Immunoanalyse, Laboratoire d’Immuno-Allergie Alimentaire (LIAA), INRA, CEA, Université Paris Saclay, DRF/Institut Joliot/SPI-Bat 136, CEA de Saclay, 91191 Gif sur Yvette Cedex, France; 3 Faculty of Public Health, Slovak Medical University, Limbová 12, 83303 Bratislava, Slovakia; 4 BioMath GmbH, Friedrich-Barnewitz-Str. 8, 18119 Rostock-Warnemünde, Germany; 5 Institute for Biosafety in Plant Biotechnology, Julius Kühn-Institut, Federal Research Centre for Cultivated Plants, Erwin-Baur-Str. 27, 06484 Quedlinburg, Germany; 6 Institute for Food Toxicology and Analytical Chemistry, University of Veterinary Medicine Hannover, Bischofsholer Damm 15, 30173 Hannover, Germany; 7 Present Address: Biostatistics (340c), University of Hohenheim, Fruwirthstr. 23, 70599 Stuttgart, Germany; 8 Present Address: Max Rubner-Institut, Federal Research Institute of Nutrition and Food, Haid-und-Neu-Str. 9, 76131 Karlsruhe, Germany

**Keywords:** Acquired immunity analysis, Anti-Cry1Ab antibodies, Anti-maize protein antibodies, Cellular immune response, Cry1Ab, Food allergenicity, Genetically modified maize MON810, GRACE, Humoral immune response, Immune cell phenotyping, Native immunity analysis, OECD Test Guideline no. 408—repeated dose 90-day oral toxicity study in rodents (1998)

## Abstract

The genetically modified maize event MON810 expresses a *Bacillus thuringiensis*-derived gene, which encodes the insecticidal protein Cry1Ab to control some lepidopteran insect pests such as the European corn borer. It has been claimed that the immune system may be affected following the oral/intragastric administration of the MON810 maize in various different animal species. In the frame of the EU-funded project GRACE, two 90-day feeding trials, the so-called studies D and E, were performed to analyze the humoral and cellular immune responses of male and female Wistar Han RCC rats fed the MON810 maize. A MON810 maize variety of Monsanto was used in the study D and a MON810 maize variety of Pioneer Hi-Bred was used in the study E. The total as well as the maize protein- and Cry1Ab-serum-specific IgG, IgM, IgA and IgE levels, the proliferative activity of the lymphocytes, the phagocytic activity of the granulocytes and monocytes, the respiratory burst of the phagocytes, a phenotypic analysis of spleen, thymus and lymph node cells as well as the in vitro production of cytokines by spleen cells were analyzed. No specific Cry1Ab immune response was observed in MON810 rats, and anti-maize protein antibody responses were similar in MON810 and control rats. Single parameters were sporadically altered in rats fed the MON810 maize when compared to control rats, but these alterations are considered to be of no immunotoxicological significance.

**Electronic supplementary material:**

The online version of this article (10.1007/s00204-018-2230-z) contains supplementary material, which is available to authorized users.

## Introduction

The genetically modified (GM) maize event MON810 expresses a *Bacillus thuringiensis*-derived gene, namely, a truncated *cry1Ab* gene encoding an insecticidal protein (δ-endotoxin; Schnepf et al. 1998), to control some lepidopteran insect pests such as the European corn borer (*Ostrinia nubilalis*; Hill et al. 1995). Concerns regarding potential adverse health effects following the ingestion of the MON810 maize have been raised, and it has been claimed that the immune system in Atlantic salmon (Sagstad et al. 2007; Gu et al. 2013), mice (Finamore et al. 2008; Adel-Patient et al. 2011) and pigs (Walsh et al. 2011) may be affected following the oral/intragastric administration of the MON810 maize. Although feeding Wistar rats with a powder diet containing 60% Bt rice for 90 days did not induce the anti-Cry1Ab IgG and IgE antibody production in the animals, feeding Wistar rats with the powder diet containing 60% Bt rice spiked with 0.1% of purified Cry1Ab for 28 days led to the detection of low levels of anti-Cry1Ab-specific IgG antibodies, but not to detectable levels of IgE antibodies (Kroghsbo et al. 2008). Kroghsbo et al. (2008) suggested that exposure via inhalation, not ingestion, induced Cry1Ab-specific immune responses in Wistar rats, since the diet was given to the animals as a powdered preparation, which can easily be inhaled. In this context, Guerrero et al. (2004, 2007) reported immunogenic effects of Cry1Ab applied via the intranasal route. This is in line with a study by Andreassen et al. (2015a), which showed that the intranasal administration of purified Cry1Ab resulted in the production of anti-Cry1Ab-specific IgG1 and IgE antibodies in BALB/c mice.

A key objective of the GRACE (GMO Risk Assessment and Communication of Evidence; http://www.grace-fp7.eu) project funded by the European Commission within the 7th Framework Programme was to conduct 90-day animal feeding trials, animal studies with an extended time frame as well as analytical, in vitro and in silico studies on GM maize, to provide recommendations on the appropriateness of these tools for the risk assessment of GM crops by considering the scientific strengths and limitations of the different approaches. For this purpose, the GM maize variety MON810 was chosen. The authors underline that the GRACE project was not expected to provide data for the reassessment of the safety profile of the MON810 maize variety, but to explore the value of different approaches including studies on the humoral and cellular immune responses in the context of the EU regulation for the risk assessment of whole GM food/feed.

In the frame of the GRACE project, two 90-day feeding trials, the so-called studies D and E, were performed to analyze the humoral and cellular immune responses of male and female Wistar Han RCC rats to the MON810 maize, whereby a MON810 maize variety of Monsanto was used in the study D and a MON810 maize variety of Pioneer Hi-Bred was used in the study E.

In the present study, the total as well as the Cry1Ab-specific and maize protein serum antibody levels were measured, thereby allowing to determine whether Cry1Ab could potentially elicit an immunogenic and/or adjuvant effect in Wistar rats under the chosen experimental conditions. Moreover, the proliferative activity of the lymphocytes, the phagocytic activity of the granulocytes and monocytes, the respiratory burst of the phagocytes, a phenotypic analysis of spleen, thymus and lymph node cells as well as the in vitro production of cytokines by spleen cells were analyzed.

## Materials and methods

### Maize varieties and diets

The feeding trials D and E performed in the frame of the GRACE project used the same batch of diets as studies A and B (Zeljenková et al. 2014), respectively, but the diets were further stored for 10 months at − 21 °C. The maize varieties and the diets used are listed in Table 1. A MON810 maize variety of Monsanto was used in the study D and a MON810 maize variety of Pioneer Hi-Bred was used in the study E. The MON810 event content in the diets containing 11 and 33% of the GM MON810 maize at the DNA and the protein level as well as the average daily amount of Cry1Ab ingested by the rats are shown in Table 2. The diets containing the conventional maize varieties PR33W82 and PR32T83 contained very low levels of the MON810 maize event (Table 2), consistent with the detection of MON810 in the maize batches used as input material for these diets (Zeljenková et al. 2014).

**Table 1:** Maize variety content of the different diets used in the rat feeding trials D and E

Diet	Maize variety content (%)
Feeding trial D	
33% near-isogenic non-GM maize	33% DKC6666^a^
11% GMO	11% DKC6667-YG^b^ + 22% DKC6666
33% GMO	33% DKC6667-YG
Feeding trial E	
33% near-isogenic non-GM maize	33% PR32T16^c^
11% GMO	11% PR33D48^d^ + 22% PR32T16
33% GMO	33% PR33D48

^a^Near-isogenic maize variety of DKC6667 YG, from Monsanto

^b^Transgenic maize variety (MON 810), from Monsanto

^c^Near-isogenic maize variety of PR33D48, from Pioneer Hi-Bred

^d^Transgenic maize variety (MON 810), from Pioneer Hi-Bred

**Table 2:** Cry1Ab levels in the different diets used in the rat feeding trials D and E

Study D	Control	11% GMO	33% GMO
	33% DKC6666	11% DKC6667-YG + 22% DKC6666	33% DKC6667-YG
MON810 maize event—genetically modified DNA (%)	Detected, n.q.	14.6	50.8
Cry1Ab (ng/mg protein)	Not detected	0.77	2.83
Average amount of ingested Cry1Ab (µg/rat/day)			
Males	–	3	11
Females	–	2	8

Study E	Control	11% GMO	33% GMO
	33% PR32T16	11% PR33D48 + 22% PR32T16	33% PR33D48
MON810 maize event—genetically modified DNA (%)	Not detected	18.9	47.6
Cry1Ab (ng/mg protein)	Not detected	2.01	5.15
Average amount of ingested Cry1Ab (µg/rat/day)			
Males	–	7	17
Females	–	5	13

*n.q*. not quantifiable

### Rat feeding trials

The 90-day feeding trials D and E were performed at the animal housing facility of the Slovak Medical University (Bratislava, Slovakia) by taking into account the guidance for such studies published by the EFSA Scientific Committee in 2011 (EFSA Scientific Committee 2011) and the OECD Test Guideline 408 (OECD 1998). For this purpose, 5-week-old male and female Wistar Han RCC rats were purchased from Harlan (San Pietro al Natisone, Italy). The study design, the performance and the results of the feeding trials D and E including the periodical health status observations, the haematology, clinical biochemistry, gross necropsy and histopathology findings as well as the corresponding statistical analyses have previously been published (Schmidt et al. 2015, 2016). These data are freely accessible via https://www.cadima.info/.

### Assessment of the humoral immune response

The humoral immune response was analyzed at the Laboratoire d’Immuno-Allergie Alimentaire of the Institut National pour la Recherche Agronomique (INRA, Gif sur Yvette Cedex, France).

#### Proteins, antibodies and reagents

Enzyme immunometric assays were performed in 96-well microtiter plates (Immunoplate Maxisorb^®^, Nunc, Roskilde, Denmark) using the AutoPlate Washer and Microfill Microplate Dispenser equipment from BioTek Instruments (Avantec, Rungis, France).

Maize flour from near-isogenic non-GM maize (DKC6666) and MON810 maize (DKC6667-YG) was suspended in 50 mM carbonate buffer (pH 9.6) containing 0.05% Tween and 0.05% dithiothreitol (DTT). For the protein extraction, 4 ml of extraction buffer per 500 mg of maize flour were used. After an incubation at 20 °C for 2 h on a rotational shaker, extracts were centrifuged (1000×*g*, 15 min, 4 °C). The lipid layer was removed and the supernatants containing the extracted proteins were collected and dialyzed against the 50 mM carbonate buffer (pH 9.6) to remove Tween and DTT. The total protein content of the extract was quantified using the BCA kit and following the instructions of the manufacturer (Pierce, Thermo Scientific, Rockford, IL, USA). Recombinant Cry1Ab protoxin was produced in *Escherichia coli* JM103 carrying the expression vector pKK223-3:cry1Ab (kindly provided by D. R. Zeigler, Bacillus Genetic Stock Center, Ohio State University, Columbus, OH) and cleaved with trypsin to yield the Cry1Ab toxin (Miranda et al. 2001; Guimaraes et al. 2010). The obtained Cry1Ab toxin was characterized by electrophoresis, mass spectrometry and specific monoclonal antibodies produced in the lab, as previously described (Guimaraes et al. 2010; Adel-Patient et al. 2011). No endotoxin was detected in protoxin/toxin preparations, as determined by the Lumulus Amebocyte Lysate test (Sigma-Aldrich Chimie, Lyon, France).

To validate the immunoassays and to produce an internal standard for anti-maize-/anti-Cry1Ab-specific Ab immunoassays, plasma from naïve Wistar Han RCC rats as well as from non-GM maize-, MON810 maize- and Cry1Ab-immunized 6-week-old female Wistar Han RCC rats was obtained. Rats were purchased from the Harlan Laboratories (Gannat, France) and bred under standard SPF conditions (autoclaved bedding and sterile water). All animal experiments were performed according to European Community rules of animal care and with authorization no. 91-368 of the French Veterinary Services. All experiments were covered by agreement No. 2009-DSV-074 from the Veterinary Inspection Department of Essonne (France). Rats were acclimated for 2 weeks before experimentation and were then kept naïve or were immunized with near-isogenic non-GM maize (DKC6666), MON810 maize (DKC6667-YG) or purified Cry1Ab protoxin (*n* = 3 rats/group). The Cry1Ab protoxin was previously shown to be highly immunogenic (Adel-Patient et al. 2011). Immunization was performed via the i.p. route using 100 µg of purified Cry1Ab or 500 µg of protein extracted from the maize varieties and alum as adjuvant (Alhydrogel 2%, Eurobio, Les Ulis, France). After three administrations 2 weeks apart, rats were anesthetized with Isoflurane Belamont (Nicholas Piramal Ltd., London, UK) and blood obtained via aorta puncture. After a centrifugation at 1200×*g* for 20 min, plasma samples were aliquoted and kept at − 20 °C until used. A plasma pool per group was also prepared.

#### Development of immunoassays to measure total IgG, IgE, IgA and IgM levels in rat plasma

Total antibodies were assessed as two-site immunometric assays using a first specific antibody as capture antibody and a second labelled antibody as tracer. Commercial antibodies were selected based on availability and literature (Table 3), and were tested as capture antibodies or biotinylated to be used as tracer antibodies.

**Table 3:** Antibodies tested for the development of the immunoassays

Antibody	Origin	Type/clone	Reference no./source
Anti-rat IgA heavy chain	Mouse	Monoclonal MARA-1	MCA191/AbD Serotec
Anti-rat IgM	Mouse	Monoclonal MARM-4	MCA189/AbD Serotec
Anti-rat IgE	Mouse	Monoclonal MARE-1	MCA193/AbD Serotec
Anti-rat IgE	Sheep	Polyclonal	STAR109/AbD Serotec
Anti-rat IgG F(c)	Rabbit	Polyclonal	OARA05389/Aviva Systems Biology
Anti-rat IgG	Goat	Polyclonal	STAR71/AbD Serotec
Anti-rat κ/λ	Mouse	Monoclonal MARK-1/MARL-15	SUN202/AbD Serotec

In a first step, commercial standard isotypes were directly immobilized to test the specificity/cross-reactivity and functionality of each biotinylated antibody and to select the antibodies to be further used. Various concentrations of standard isotypes (IgA kappa clone IR22, IgE kappa clone IR162, IgM kappa clone IR473 and polyclonal IgG, purified from rat sera; all from Bio-Rad AbD Serotec, Oxford, UK) were then passively immobilized onto microtiter plates (0.01 to 1 µg/ml, 50 mM phosphate buffer pH 7.4, 100 µl/well; 18 h at + 4 °C). After washing the microtiter plates (washing buffer: 0.01 M phosphate buffer, pH 7.4, containing 0.05% Tween 20) and saturation in EIA buffer (0.1 M phosphate buffer, 0.1% bovine serum albumin, 0.15 M NaCl, 0.01% sodium azide), 100 µl of biotinylated antibodies (EZ-Link Sulfo-NHS-LC-Biotin; Pierce, Rockford, IL; biotin:antibody molar ratio = 20) were added to the microtiter plates (10–100 ng/ml in EIA buffer) and incubated for 3 h at 20 °C. After washing, acetylcholinesterase (AChE)-labelled streptavidin was added for 15 min at 20 °C. Microtiter plates were then extensively washed and solid phase-bound AChE activity was determined by addition of 200 µl/well of Ellman’s reagent as an enzyme substrate and the absorbance was measured at 414 nm (Pradelles et al. 1985) using automatic reader plates (MultiskanEx, Thermo Electron Corporation, Vantaa, Finland). Results are expressed as absorbance unit at 414 nm (mAbs414_nm_).

Pre-selected antibodies and concentrations were then used in sandwich immunoassays. Different anti-isotype antibodies were passively immobilized for 18 h at 4 °C (5 µg/ml in 50 mM phosphate buffer pH 7.4, 100 µl/well). After washing and saturation in EIA buffer, standard isotypes were added (0–100 ng/ml, in EIA buffer) for 18 h at 4 °C. After extensive washing, biotinylated antibodies were added and the binding of the antibodies was evaluated as mentioned before. Specificity/cross-reactivity was assessed by testing the different standard isotypes in each specific assay, while intra- and inter-assay variability was assessed by reproducing the same assay on different plates on the same day or on two separate days (1 week apart). In a final step, to assess the parallel course of the curves for the standards and the plasma samples, plasma samples from naïve and immunized rats were tested using serial dilutions in parallel to the isotype standard. Nonspecific binding (NSB) was determined using dilution buffer instead of standard/serum.

#### Development of immunoassays to measure maize protein and Cry1Ab-specific IgG, IgE, IgA and IgM levels in rat plasma

Specific antibodies were assessed on plates coated with maize protein extracts or purified Cry1Ab (5 µg/ml, diluted in 50 mM phosphate buffer pH 7.4, 100 µl/well). For validation, serial dilutions of individual plasma samples/pool plasma from naïve or immunized rats were performed in EIA buffer and applied to coated plates for 18 h at 4 °C. After washing, biotinylated antibodies were applied for 3 h at room temperature and the binding of the antibodies was evaluated as mentioned before. No anti-maize-specific IgE or IgA was detected in the plasma from maize-immunized rats and no anti-Cry1Ab-specific antibodies were detected in rats immunized with MON810 maize. Intra and inter-assay variabilities were assessed as mentioned before. Standard curves for anti-maize- or anti-Cry1Ab-specific IgG and anti-maize- or anti-Cry1Ab-specific IgM were plotted using serial dilutions of the corresponding plasma from immunized rats (8 points). An arbitrary value of 100 (100 AU) was assigned to pooled sera from maize protein-/Cry1Ab-immunized rats diluted 1/1000 (IgM) or 1/10,000 (IgG).

#### Assessment of total and specific antibodies in plasma from the maize-fed rats in trials D and E

After optimization, validation and preliminary experiments with randomly selected maize-fed rat plasma samples (*n* = 10), immunoassays for total and specific antibodies were performed with the plasma samples from the different rat groups in trials D and E. Standard curves were included in each microtiter plate and all samples were analyzed in a randomized and blinded manner. All analyses were repeated once. For total antibodies, internal controls (IC, pool of sera from naive/immunized rats) were also included in each microtiter plate.

### Assessment of the cellular immune response

The cellular immune response was analyzed at the Slovak Medical University (Bratislava, Slovakia).

#### Phenotypic analysis of spleen, mesenteric lymph nodes, bone marrow and thymus

Nine microliters of a mixture of labelled monoclonal antibodies were added to 90 µl of a spleen, mesenteric lymph node, bone marrow and thymus cell suspension (2 × 10^6^ cells/ml) and incubated for 30 min at 4 °C in the dark. The following antibodies, all purchased from eBioscience (San Diego, CA), were used to stain the cells: Anti-Rat CD3 FITC, Anti-Rat CD4 PE, Anti-Rat CD8a PerCP-eFluor 710, Anti-Rat CD45R PE and Anti-Rat CD161 PerCP-eFluor 710. Isotype controls (Mouse IgG3 Isotype Control-FITC, Mouse IgG2a K Isotype Control-PE, Mouse IgG1 K Isotype Control-PerCP-eFluor 710 and Mouse IgG2b K Isotype Control-PE) were used as negative controls to determine background fluorescence. Red blood cells were lysed with a lysis solution for 15 min and, finally, phosphate-buffered saline (PBS) was added. The lysis solution was prepared by adding 0.829 g NH_4_Cl (Lachema, Brno, Czech Republic), 0.1 g KHCO_3_ and 0.0037 g Na_2_EDTA (both from Sigma-Aldrich) to 100 ml aqua ad injectionem (Imuna Pharm, Šarišské Michaľany, Slovakia). Samples were analyzed using an EPICS XL flow cytometer (Beckman Coulter). The percentage of CD3^+^, CD3^+^CD4^+^, CD3^+^CD8a^+^, CD3^−^CD161^+^ and CD3^−^CD45R^+^ cells in each sample was measured in duplicates and by making use of forward and side scatter gates.

#### Phagocytic activity of granulocytes and monocytes, and respiratory burst of phagocytes

Thirty microliters of rat heparinized whole blood were pipetted into a tube and 10 µl of a working solution of hydroethidine (dihydroethidium bromide, HE; Polysciences, Warrington, PA) were then added. The HE working solution was prepared by adding 10 µl of HE stock solution (15.75 mg HE in 5 ml dimethylformamide; Merck, Kenilworth, NJ) to 1 ml Medium 199 (Gibco, Invitrogen, Paisley, UK). Samples were incubated for 15 min at 37 °C. Three microliters of fluorescein-labelled *Staphylococcus aureus* bacteria (Molecular Probes, Eugene, OR) was added to each of the “test” tubes (1.4 × 10^6^ bacteria per test). All tubes were incubated for another 15 min at 37 °C. Samples were put on ice and 700 µl of the cold lysis solution described above were added for 10 min to lyse red blood cells. In the case of the “control” tubes, the *Staphylococcus aureus* bacteria were added after the lysis solution. Samples were analyzed using an EPICS XL flow cytometer (Beckman Coulter). The percentage of phagocytic monocytes and phagocytic granulocytes (i.e. those that had phagocytised the fluorescein-labelled *Staphylococcus aureus* bacteria) and the percentage of granulocytes with respiratory burst (i.e., those in which dihydroethidine was converted to a fluorescent metabolite by reactive oxygen species) were measured in duplicates and by making use of forward and side scatter gates.

#### Proliferative activity of lymphocytes

The spleen was removed using sterile instruments and placed in sterile RPMI 1640 culture medium with L-glutamine and HEPES buffer (Sigma-Aldrich, St. Louis, MO) supplemented with 5 IU heparin/ml (Zentiva, Prague, Czech Republic) and 12 µg gentamycin/ml (Sandoz, Basel, Switzerland). Spleen cells were obtained under sterile conditions by washing the spleen with RPMI medium and by making use of a syringe with a needle. The spleen cell suspension was centrifuged at 130×*g* for 15 min and resuspended in complete RPMI medium containing 10% foetal calf serum (FCS, PAA, Linz, Austria). Cell suspensions (2 × 10^6^ cells/ml) were dispensed in triplicate wells (150 µl/well) of a 96-well microtiter culture plate. The mitogens, all purchased from Sigma-Aldrich, were added at the following final concentrations: concanavalin A (Con A; 2.5 µg/ml), phytohemagglutinin (PHA; 25 µg/ml) and pokeweed mitogen (PWM; 2.5 µg/ml). Moreover, recombinant Cry1Ab at final concentrations of 5, 50 and 500 ng/ml and protein extracts from near-isogenic non-GM and MON810 maize at final concentrations of 50 ng/ml, 500 ng/ml and 5 µg/ml were added to additional cell culture plates. Recombinant Cry1Ab as well as the maize protein extracts were obtained as described above. The plates with mitogens were incubated for 48 h and those with maize antigens and CryAb toxin for 6 days at 37 °C and 5% CO_2_. Thereafter, each well was pulsed with 1 µCi [^3^H]-thymidine (Moravek Biochemicals, Brea, CA) diluted in 20 µl medium and the plates were incubated at 37 °C for another 24 h. The cell cultures were then harvested on glass filtre papers and the filtres were placed in scintillation fluid (Perkin Elmer, Waltham, MA). Radioactivity was measured using a Beta Scintillation counter Microbeta 2 (Perkin Elmer). Counts per minute (cpm)/cell culture were measured in triplicates for each variable. The index was calculated as the ratio of cpm/cell culture in wells stimulated with a mitogen/a protein extract and cpm/cell culture in unstimulated wells.

#### In vitro production of cytokines

Spleen cells were incubated in complete RPMI medium as described above. One hundred and fifty microliters of the spleen cell suspension (2 × 10^6^ cells/ml) were dispensed in triplicate wells of a 96-well microtiter culture plate. The mitogen Con A (2.5 µg/ml) or the Cry 1Ab toxin (50 ng/ml) were added in a volume of 50 µl. The plates were incubated at 37 °C in the presence of 5% CO_2_ for 72 or 144 h, respectively. Thereafter, the supernatants were removed and stored at − 70 °C. The ProcartaPlex^®^ Rat Th Complete Panel (14 plex) from eBioscience was used to measure the levels of interleukin (IL)-1α, IL-1β, IL-2, IL-4, IL-5, IL-6, IL-10, IL-12p70, IL-13, IL-17A, interferon-γ (IFN-γ), granulocyte-colony-stimulating factor (G-CSF), granulocyte–macrophage colony-stimulating factor (GM-CSF) and tumour necrosis factor-α (TNF-α) in spleen cell culture supernatants by following the instructions of the manufacturer and by making use of a Luminex^®^ 200 apparatus (Luminex, Madison, WI, USA).

### Statistics

**Fig. 1:**
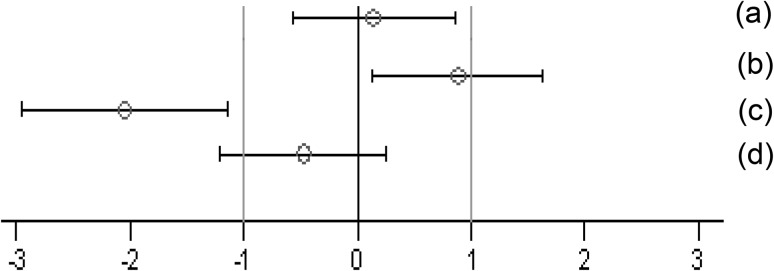
Simplified version of a graph allowing visual assessment of statistical significance as well as the supposed biological and possible toxicological relevance of group comparisons. The standard effect size point estimate (circle) and the 95% confidence limits (whiskers, bars show confidence interval) illustrate the (standardized) effect size between two groups. The vertical black line indicates no statistically significant difference (zero difference), while the vertical grey lines indicate the supposed biological and possible toxicological relevance limits (here ± 1.0 SD, according to the study design). If the confidence interval bars cross the zero line but not the grey lines (lie within the ± 1.0 limits), there is evidence for no statistical significance as well as no biological relevance (case a). Two groups are significantly different when the confidence interval bars do not cross the black vertical line (cases b, c). The effect size between two groups is supposed to be potentially relevant, when the confidence interval bars lie outside the ± 1.0 SD limits (case c). Case b indicates statistical significance, but no clear biological relevance. Case d indicates no statistical significance, but no clear negation of biological relevance. This figure is Fig. 1 of the study by Zeljenková et al. (2014)

The immunological parameters were analyzed descriptively (*N*, mean, median, standard deviation, minimum, maximum, 95% confidence interval of the mean). Standardized effect sizes (SES: difference in means between two groups divided by the pooled standard deviation [SD]) as well as their 95% confidence intervals were calculated according to Nakagawa and Cuthill (2007); for details see also Schmidt et al. (2015). The GMO groups were compared to the control groups: GMO11%-control and GMO33%-control. All immunological parameters were displayed in one SES graph and the data are expressed as the cage mean ± SD. In this paper, when comparing the different parameters between a control and a second group, the wording “significantly different” is based on the interpretation of the calculated SES estimates (Fig. 1).

## Results

### Humoral immune response

**Fig. 2:**
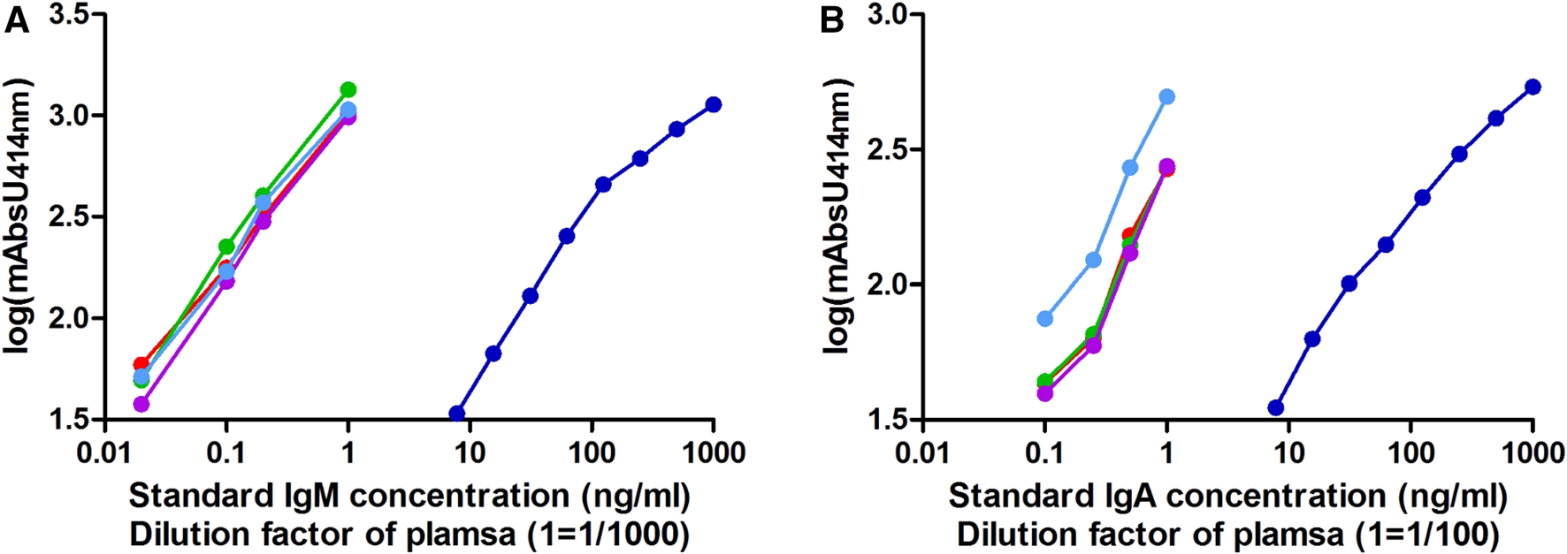
Parallelism of the curves for total IgM (**a**) and IgA (**b**) obtained with the isotype standard (dark blue) and diluted plasma from experimental rats immunized with GM maize (green), conventional maize (purple), Cry1Ab protoxin (clear blue) or kept naïve (red). The *x*-axis represents the concentration of the isotype standard or the dilution factor of the different plasma samples (a dilution factor of 1 corresponds to an initial dilution factor of 1/1000 for IgM and 1/100 for IgA). mAbs414nm, absorbance unit at 414 nm. (Color figure online)

**Fig. 3:**
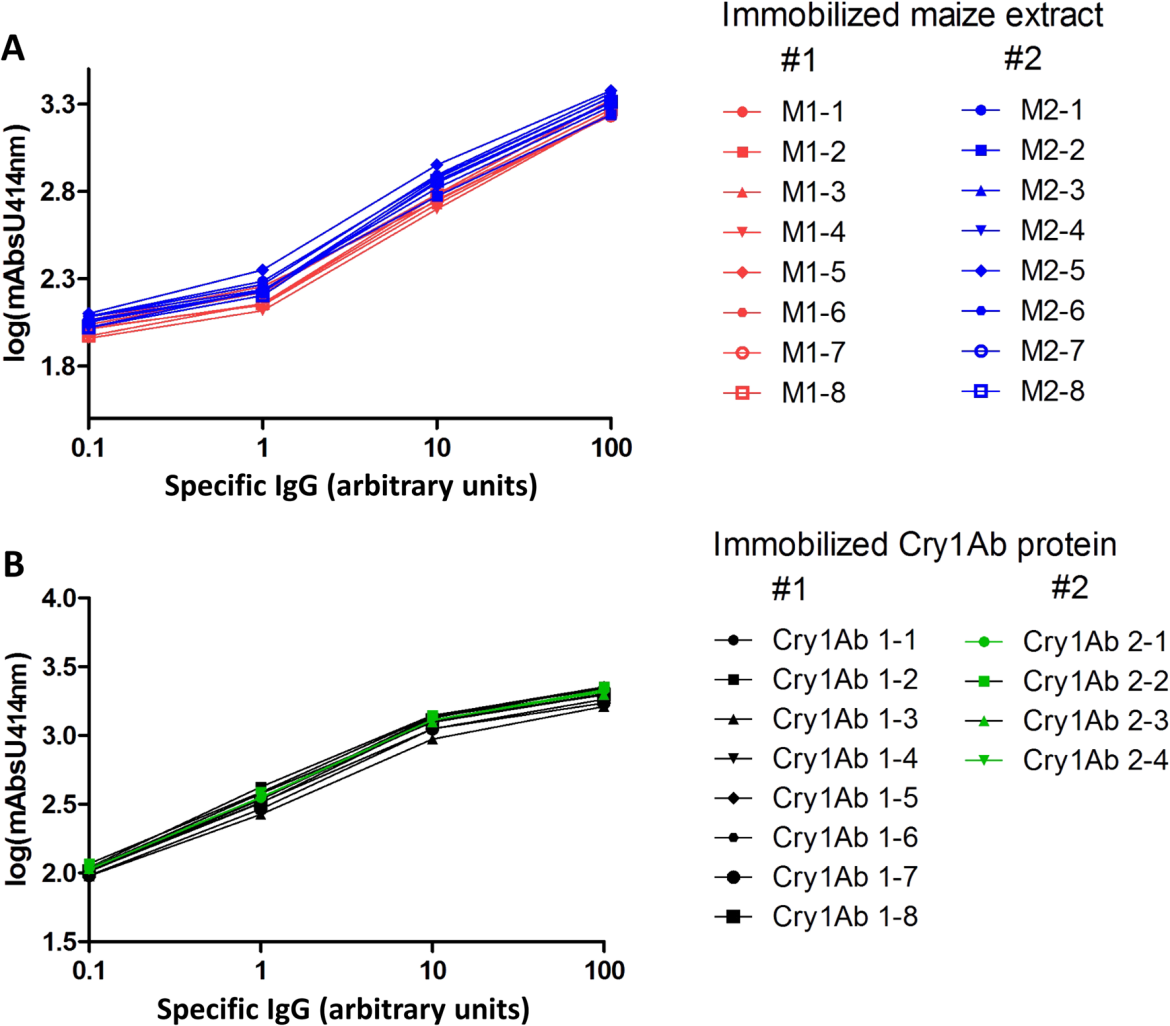
Anti-maize- (**a**) and anti-Cry1Ab-specific IgG (**b**) in plasma from rats immunized with maize protein extract or Cry1Ab, respectively. Assays were performed on the same days on up to eight separate plates. Plates were coated with extracts/protein purified on different days (series #1 and #2). The *x*-axis represents arbitrary units of specific IgG; a value of 100 arbitrary units was assigned to pooled plasma from maize-/Cry1Ab-immunized rats diluted 1/10,000. mAbs414nm, absorbance unit at 414 nm

The antibodies selected for the various immunoassays are indicated in Table 4. Typical IgE standard curves showed an excellent intra- and inter-assay reproducibility (Supplementary Material, Fig. 1). Moreover, a good parallel course and reproducibility of the curves for the standards and diluted plasma were also observed for total IgM and IgA (Fig. 2), demonstrating that the calculated concentrations do not depend on the plasma dilution and the experimental day. The limits of detection, determined as the means of NSB + 3*σn* − 1, were 98 pg/ml for IgE, 69 pg/ml for IgG and 7.8 ng/ml for IgA and IgM. Specific IgG and IgM immunoassays were further validated and standardized by making use of a pool of plasma samples from rats immunized with maize or Cry1Ab. The sensitivity and reproducibility of the corresponding assays were high, as exemplarily shown for IgG in Fig. 3. Preliminary experiments using some randomly selected plasma samples from rats fed MON810 maize were performed, thereby allowing to determine the dilutions to be used for the analysis of the whole set of samples (Table 5).

**Table 4:** Antibodies selected for the total and specific IgE, IgG, IgA and IgM immunoassays

Isotype	Antibodies for the total immunoglobulin immunoassays		Antibodies for the specific immunoglobulin immunoassays
	Capture antibody	Labelled antibody	Labelled antibody
IgE	STAR109	MARE-1 (100 ng/ml)	MARE-1 (100 ng/ml)
IgG	STAR71	STAR71 (50 ng/ml)	STAR71 (50 ng/ml)
IgA	MARA-1	Anti-rat κ/λ (50 ng/ml)	MARA-1 (50 ng/ml)
IgM	MARM-4	Anti-rat κ/λ (50 ng/ml)	MARM-4 (50 ng/ml)

**Table 5:** Dilutions used for the determination of the total, anti-maize-specific and anti-Cry1Ab-specific antibody levels in rat plasma

Isotype	Dilutions used for the determination of		
	Total antibody levels	Anti-maize-specific antibody levels	Anti-Cry1Ab-specific antibody levels
IgE	1/20 and 1/40	1/10	1/10
	Standard from 400 ng/ml (SDF = 4)		
IgG	1/2 × 10^6^ and 1/10^7^	1/100 and 1/500	1/50 and 1/100
	Standard from 30 ng/ml (SDF = 3)		
IgA	1/100 and 1/500	1/10	1/10
	Standard from 1000 ng/ml (SDF = 2)		
IgM	1/2000 and 1/10,000	1/40	1/40
	Standard from 1000 ng/ml (SDF = 2)		

*SDF* Serial Dilution Factor (standard curves were performed with 8 concentration points)

The amount of total IgG, maize-specific IgG and total IgE measured in the plasma of male and female rats in the feeding trials D and E are shown in Table 6. In study D, the total plasma IgG, anti-maize-specific IgG and total IgE levels in male rats fed the control or GMO diets were similar (i.e. the differences between the groups were not statistically significant) and this was also the case of the female animals. In study E, the anti-maize-specific IgG level in plasma of male rats fed the 11% GMO diet was significantly higher than that in plasma of male rats fed the control diet, but this was not the case in male rats fed the 33% GMO diet (Table 6). All other parameters were similar in male and female rats. The anti-maize protein-specific IgE and IgA antibody levels were below the detection limit in all experimental groups. No anti-Cry1Ab-specific antibodies were detected in any group.

**Table 6:** Total IgG, anti-maize protein-specific IgG and total IgE levels in plasma of male and female rats in feeding trials D and E

Parameter	Male rats			Female rats		
	Control	11% GMO	33% GMO	Control	11% GMO	33% GMO
Trial D						
Total IgG level (mg/ml)	2.47 ± 0.73	1.95 ± 0.40	2.56 ± 0.36	3.05 ± 0.50	2.73 ± 0.61	2.77 ± 0.53
Anti-maize protein-specific IgG level (arbitrary units/ml)	985 ± 105	1149 ± 157	971 ± 303	1006 ± 342	879 ± 148	1130 ± 197
Total IgE level (ng/ml)	38.07 ± 13.48	42.03 ± 14.76	57.72 ± 33.71	61.66 ± 28.68	47.42 ± 15.89	49.98 ± 29.79
Trial E						
Total IgG level (mg/ml)	2.32 ± 1.06	2.42 ± 0.67	2.85 ± 0.27	2.97 ± 0.83	3.27 ± 0.49	2.92 ± 0.46
Anti-maize protein-specific IgG level (arbitrary units/ml)	900 ± 221	1287 ± 150*	1682 ± 1210	1294 ± 283	970 ± 163	1234 ± 399
Total IgE level (ng/ml)	38.81 ± 15.07	46.63 ± 26.94	41.16 ± 10.62	66.02 ± 24.06	42.63 ± 18.22	42.37 ± 13.45

The results are expressed as cage mean ± SD (five cages, *n* = 10 rats)

*Statistically significant difference to the control value based on the 95% confidence interval of the SES

### Cellular immune response

The phenotypic analysis of spleen, mesenteric lymph node, bone marrow and thymus cells of male and female rats in the feeding trial D is shown in Table 7. The percentage of CD3^+^CD4^+^ cells in the thymus of male rats fed the 33% GMO diet was significantly lower than that of the corresponding control group, whereas no statistically significant differences regarding all other cell phenotypes and lymphoid organs between rats fed the control and the GMO diets were observed. The phenotypic analysis of spleen, mesenteric lymph node, bone marrow and thymus cells of male and female rats in the feeding trial E is shown in Table 8. The percentages of CD3^+^ and CD3^+^CD4^+^ cells in the spleen of female rats fed the 33% GMO diet were significantly higher and, concomitantly, the percentage of CD3^−^ cells in the spleen of female rats was significantly lower than that of the control group, whereas no statistically significant differences regarding all other cell phenotypes between rats fed the control and the GMO diets were observed.

**Table 7:** Phenotypic analysis of spleen, lymph node, bone marrow and thymus cells of male and female rats in the feeding trial D

Parameter	Male rats			Female rats		
	Control	11% GMO	33% GMO	Control	11% GMO	33% GMO
Spleen CD3^+^CD8^+^ cells	40.72 ± 8.26	37.35 ± 4.65	35.94 ± 5.99	42.11 ± 4.80	39.68 ± 5.72	37.87 ± 5.85
Spleen CD3^+^ cells	59.51 ± 8.82	55.97 ± 4.07	56.68 ± 5.83	64.72 ± 3.17	63.39 ± 7.69	60.68 ± 6.23
Spleen CD3^−^ cells	40.50 ± 8.82	44.03 ± 4.07	43.32 ± 5.83	35.29 ± 3.17	36.61 ± 7.69	39.33 ± 6.23
Spleen CD3^+^CD4^+^ cells	40.48 ± 7.16	37.20 ± 3.93	34.09 ± 7.79	42.49 ± 4.52	39.70 ± 6.14	38.05 ± 4.96
Spleen CD3^−^CD45R^+^ cells	24.16 ± 4.92	29.96 ± 2.17	28.21 ± 3.50	24.66 ± 2.73	25.40 ± 3.48	26.89 ± 5.03
Spleen CD3^−^CD161^+^ cells	19.62 ± 3.63	24.47 ± 2.62	23.44 ± 2.53	18.97 ± 7.95	18.97 ± 6.89	20.62 ± 7.88
Lymph node CD3^+^CD8^+^ cells	28.87 ± 12.57	29.36 ± 17.70	27.93 ± 13.85	41.73 ± 5.70	40.02 ± 5.87	41.62 ± 6.01
Lymph node CD3^+^ cells	41.89 ± 19.48	42.35 ± 20.81	43.97 ± 19.53	56.51 ± 8.53	53.96 ± 9.41	55.92 ± 8.47
Lymph node CD3^−^ cells	58.12 ± 19.48	57.65 ± 20.81	56.03 ± 19.53	43.49 ± 8.53	46.04 ± 9.41	44.08 ± 8.47
Lymph node CD3^+^CD4^+^ cells	26.22 ± 10.05	26.62 ± 16.46	24.46 ± 12.00	38.88 ± 4.09	37.72 ± 4.75	39.03 ± 4.96
Lymph node CD3^−^CD45R^+^ cells	52.81 ± 14.43	53.98 ± 16.52	56.80 ± 16.19	40.96 ± 6.49	44.44 ± 6.11	41.08 ± 7.88
Bone marrow CD3^+^ cells	7.75 ± 1.05	7.25 ± 3.11^a^	8.22 ± 2.73	17.44 ± 6.17	15.63 ± 5.71	13.27 ± 2.38
Bone marrow CD3^−^ cells	92.26 ± 1.05	92.76 ± 3.11^a^	91.79 ± 2.73	82.57 ± 6.17	84.38 ± 5.71	86.74 ± 2.38
Bone marrow CD3^−^CD45R^+^ cells	71.60 ± 7.46	73.45 ± 8.12^a^	70.37 ± 7.28	64.84 ± 12.22	61.87 ± 16.91	61.91 ± 20.71
Bone marrow CD3^−^CD161^+^ cells	12.77 ± 6.32	13.43 ± 5.25^a^	12.54 ± 5.77	15.55 ± 4.55	13.19 ± 4.20	14.80 ± 5.09
Thymus CD3^+^CD8^+^ cells	21.37 ± 2.63	22.92 ± 3.95	17.57 ± 4.12	19.46 ± 2.67	20.57 ± 3.20	20.49 ± 3.44
Thymus CD3^+^ cells	25.11 ± 5.60	27.22 ± 7.77	21.42 ± 7.41	21.73 ± 2.73	22.90 ± 3.42	22.81 ± 3.74
Thymus CD3^−^ cells	74.90 ± 5.60	72.78 ± 7.77	78.59 ± 7.41	78.27 ± 2.73	77.11 ± 3.42	77.19 ± 3.74
Thymus CD3^+^CD4^+^ cells	20.04 ± 1.31	21.01 ± 2.42	16.40 ± 2.65*	18.95 ± 2.51	19.96 ± 3.05	19.87 ± 3.29

The table lists the percentage of cells with the indicated phenotype, expressed as cage mean ± SD (five cages; *n* = 10 rats, if not otherwise stated)

*Statistically significant difference to the control value based on the 95% confidence interval of the SES

^a^*n* = 9

**Table 8:** Phenotypic analysis of spleen, lymph node, bone marrow and thymus cells of male and female rats in the feeding trial E

Parameter	Male rats			Female rats		
	Control	11% GMO	33% GMO	Control	11% GMO	33% GMO
Spleen CD3^+^CD8^+^ cells	41.51 ± 6.81	41.41 ± 7.60	37.88 ± 9.13	39.41 ± 3.96	42.92 ± 5.48	46.58 ± 5.59
Spleen CD3^+^ cells	60.86 ± 6.82	60.66 ± 8.39	57.16 ± 11.66	62.73 ± 2.76	65.37 ± 2.10	69.69 ± 4.20*
Spleen CD3^−^ cells	39.15 ± 6.82	39.35 ± 8.39	42.84 ± 11.66	37.27 ± 2.76	34.63 ± 2.10	30.32 ± 4.20*
Spleen CD3^+^CD4^+^ cells	41.62 ± 5.79	41.00 ± 6.27	37.32 ± 8.68	39.27 ± 3.38	43.36 ± 4.96	46.75 ± 4.68*
Spleen CD3^−^CD45R^+^ cells	24.56 ± 3.99	25.86 ± 3.83	26.09 ± 5.23	25.46 ± 2.21	24.10 ± 1.62	21.21 ± 3.63
Spleen CD3^−^CD161^+^ cells	19.97 ± 3.00	21.46 ± 2.74	21.39 ± 4.25	19.25 ± 8.71	17.59 ± 7.70	16.26 ± 6.40
Lymph node CD3^+^CD8^+^ cells	33.47 ± 17.51	27.62 ± 15.23	27.29 ± 13.70^a^	42.50 ± 1.96	42.52 ± 5.80	43.69 ± 12.98
Lymph node CD3^+^ cells	45.32 ± 20.51	39.68 ± 19.83	42.93 ± 20.25^a^	57.40 ± 3.39	56.51 ± 7.44	58.01 ± 16.84
Lymph node CD3^−^ cells	54.69 ± 20.51	60.33 ± 19.84	57.08 ± 20.25^a^	42.60 ± 3.40	43.50 ± 7.44	41.99 ± 16.84
Lymph node CD3^+^CD4^+^ cells	31.44 ± 15.77	25.98 ± 12.94	23.91 ± 10.76^a^	40.35 ± 2.16	40.79 ± 5.49	41.15 ± 11.03
Lymph node CD3^−^CD45R^+^ cells	47.83 ± 14.58	52.53 ± 14.22	50.88 ± 13.95^a^	38.49 ± 2.57	39.01 ± 5.15	37.67 ± 11.65
Bone marrow CD3^+^ cells	9.27 ± 1.24	11.93 ± 5.95	9.80 ± 2.63	15.44 ± 3.91	12.30 ± 3.58	17.34 ± 5.72
Bone marrow CD3^−^ cells	90.73 ± 1.24	88.08 ± 5.94	90.21 ± 2.63	84.57 ± 3.91	87.70 ± 3.58	82.67 ± 5.73
Bone marrow CD3^−^CD45R^+^ cells	69.67 ± 6.22	65.94 ± 5.58	67.79 ± 10.36	65.92 ± 12.48	70.51 ± 11.47	68.53 ± 7.50
Bone marrow CD3^−^CD161^+^ cells	12.73 ± 7.10	11.95 ± 5.63	11.69 ± 4.43	13.22 ± 3.18	13.49 ± 4.01	13.99 ± 2.73
Thymus CD3^+^CD8^+^ cells	22.71 ± 5.24	24.17 ± 5.89	22.27 ± 4.94	22.80 ± 1.41	20.59 ± 3.26	22.42 ± 0.81
Thymus CD3^+^ cells	26.68 ± 8.54	28.11 ± 8.19	26.32 ± 8.17	25.24 ± 1.43	22.81 ± 3.36	24.82 ± 0.85
Thymus CD3^−^ cells	73.32 ± 8.54	71.90 ± 8.19	73.68 ± 8.17	74.76 ± 1.43	77.20 ± 3.36	75.18 ± 0.85
Thymus CD3^+^CD4^+^ cells	21.39 ± 3.79	22.84 ± 4.88	20.62 ± 3.34	22.39 ± 1.34	20.05 ± 2.97	21.92 ± 0.99

The table lists the percentage of cells with the indicated phenotype, expressed as cage mean ± SD (five cages; *n* = 10 rats, if not otherwise stated)

*Statistically significant difference to the control value based on the 95% confidence interval of the SES

^a^*n* = 9

Table 9 lists the percentage of phagocytic monocytes and granulocytes after incubation of the cells with labelled *Staphylococcus aureus* and the percentage of phagocytes showing respiratory burst after incubation of the cells with dihydroethidine in the feeding trials D and E. The percentage of phagocytic granulocytes and the percentage of phagocytes showing a respiratory burst were significantly higher in female rats fed the 11% GMO diet than in the control group in study D, while no other statistically significant differences regarding the above-mentioned parameters between rats fed the control and the GMO diets were observed in both studies.

**Table 9:** Phagocytic activity of monocytes and granulocytes and respiratory burst in phagocytes of male and female rats in feeding trials D and E

Parameter	Male rats			Female rats		
	Control	11% GMO	33% GMO	Control	11% GMO	33% GMO
Trial D						
Phagocytic activity of monocytes	39.76 ± 11.97	38.83 ± 11.42	32.52 ± 6.33	56.17 ± 10.42	64.08 ± 15.99	48.63 ± 7.13
Phagocytic activity of granulocytes	64.72 ± 6.05	63.10 ± 2.99	59.63 ± 5.05	64.99 ± 5.01	73.37 ± 4.82*	62.93 ± 7.39
Respiratory burst in phagocytes	67.38 ± 5.57	68.39 ± 2.61	62.16 ± 5.04	67.24 ± 6.46	76.25 ± 3.82*	65.80 ± 6.84
Trial E						
Phagocytic activity of monocytes	31.14 ± 5.79	38.38 ± 11.63	32.69 ± 8.20	48.18 ± 9.98	52.57 ± 12.66	42.61 ± 10.04
Phagocytic activity of granulocytes	59.47 ± 9.20	63.35 ± 7.48	61.07 ± 6.39	57.91 ± 14.10	68.54 ± 7.14	66.39 ± 7.35
Respiratory burst in phagocytes	61.10 ± 7.69	65.31 ± 7.52	63.73 ± 7.07	62.00 ± 12.30	70.16 ± 8.29	67.93 ± 6.55

The table lists the percentage of phagocytic monocytes and granulocytes after incubation of the cells with labelled *Staphylococcus aureus* and the percentage of phagocytes showing respiratory burst after incubation of the cells with dihydroethidine, expressed as cage mean ± SD (5 cages; *n* = 10 rats)

*Statistically significant difference to the control value based on the 95% confidence interval of the SES

The proliferative response of spleen cells from male and female rats of feeding trial D after incubation with concanavalin A, phytohemagglutinin, pokeweed mitogen, Cry1Ab, the near-isogenic non-GM maize protein extract and the GM maize protein extract is shown in Table 10. The proliferative response of spleen cells from male rats fed the 33% GMO diet when incubated with phytohemagglutinin was significantly lower than that of spleen cells from male rats fed the control diet, whereas all other proliferative responses did not differ between the rats fed the GMO diets and those fed the control diet. The proliferative response of spleen cells from male and female rats of feeding trial E incubated with concanavalin A, phytohemagglutinin, pokeweed mitogen, Cry1Ab, the near-isogenic non-GM maize protein extract and the GM maize protein extract is shown in Table 11. The proliferative response of spleen cells from male rats fed the 11% GMO diet when incubated with phytohemagglutinin and when incubated with 5 µg/ml GM maize protein as well as the proliferative response of spleen cells from male rats fed the 33% GMO diet when incubated with 50 ng/ml Cry1Ab were significantly lower than that of spleen cells from male rats fed the control diet. These differences were not observed in female rats and all other proliferative responses did not differ between the rats fed the GMO diets and those fed the control diet.

**Table 10:** Proliferative response of spleen cells from male and female rats of feeding trial D incubated with concanavalin A, phytohemagglutinin, pokeweed mitogen, Cry1Ab, the near-isogenic non-GM maize protein extract and the GM maize protein extract

Parameter	Male rats			Female rats		
	Control	11% GMO	33% GMO	Control	11% GMO	33% GMO
IPR concanavalin A	56.96 ± 10.81	55.99 ± 14.07	50.34 ± 22.91	36.92 ± 13.48	34.94 ± 23.27	29.08 ± 16.32
IPR phytohemagglutinin	22.46 ± 3.90	24.43 ± 7.64	16.42 ± 2.74*	16.83 ± 7.02	13.39 ± 5.60	10.19 ± 3.34
IPR pokeweed mitogen	11.74 ± 1.78	13.42 ± 5.43	9.73 ± 1.99	8.81 ± 3.03	11.78 ± 3.76	7.94 ± 3.35
IPR 5 ng Cry1Ab/ml	1.14 ± 0.16	1.06 ± 0.18	0.93 ± 0.20	0.94 ± 0.12	0.86 ± 0.19	1.19 ± 0.72^a^
IPR 50 ng Cry1Ab/ml	1.09 ± 0.15	1.05 ± 0.10	0.92 ± 0.26	0.91 ± 0.13	0.90 ± 0.13	0.84 ± 0.32
IPR 500 ng Cry1Ab/ml	1.11 ± 0.25	1.11 ± 0.15	0.91 ± 0.16	0.98 ± 0.14	0.99 ± 0.09	0.93 ± 0.05
IPR 50 ng non-GM maize protein/ml	1.02 ± 0.14	1.05 ± 0.33	0.98 ± 0.35	1.01 ± 0.13	0.95 ± 0.16^a^	0.98 ± 0.34
IPR 500 ng non-GM maize protein/ml	0.84 ± 0.09	1.10 ± 0.28	0.92 ± 0.43	0.91 ± 0.10	1.05 ± 0.13	1.01 ± 0.21
IPR 5 µg non-GM maize protein/ml	0.65 ± 0.15	0.74 ± 0.09	0.73 ± 0.37	0.87 ± 0.07	0.87 ± 0.08	1.01 ± 0.26
IPR 50 ng GM maize protein/ml	0.96 ± 0.18	0.93 ± 0.21^a^	0.86 ± 0.19	0.92 ± 0.08	0.93 ± 0.18	1.05 ± 0.12
IPR 500 ng GM maize protein/ml	0.80 ± 0.11	0.93 ± 0.19	0.83 ± 0.29	0.93 ± 0.11	0.90 ± 0.10	0.90 ± 0.17
IPR 5 µg GM maize protein/ml	0.84 ± 0.14	0.87 ± 0.21	0.74 ± 0.28	0.85 ± 0.16	0.83 ± 0.12	0.73 ± 0.10

Spleen cells were incubated for 3 days with concanavalin A, phytohemagglutinin or pokeweed mitogen and for 6 days with Cry1Ab, the near isogenic non-GM maize protein extract or the GM maize protein extract in the given amounts. The table lists the indexed proliferative response (IPR) = proliferative response of stimulated cells/proliferative response of non-stimulated cells, expressed as cage mean ± SD (five cages; *n* = 10 rats, if not otherwise stated)

*Statistically significant difference to the control value based on the 95% confidence interval of the SES

^a^*n* = 9

**Table 11:** Proliferative response of spleen cells from male and female rats of feeding trial E incubated with concanavalin A, phytohemagglutinin, pokeweed mitogen, Cry1Ab, the near-isogenic non-GM maize protein extract and the GM maize protein extract

Parameter	Male rats			Female rats		
	Control	11% GMO	33% GMO	Control	11% GMO	33% GMO
IPR concanavalin A	69.72 ± 28.69	52.84 ± 9.36	47.57 ± 11.45	33.80 ± 19.33	34.77 ± 19.62	38.36 ± 21.08
IPR phytohemagglutinin	27.69 ± 3.85	21.53 ± 3.06*	21.85 ± 7.39	15.71 ± 9.41	15.95 ± 11.82	18.15 ± 6.69
IPR pokeweed mitogen	14.15 ± 5.21	13.24 ± 2.75	11.93 ± 3.63	9.44 ± 4.91	9.96 ± 7.22	11.46 ± 3.85
IPR 5 ng Cry1Ab/ml	1.02 ± 0.16	1.09 ± 0.23	0.92 ± 0.19	1.93 ± 2.26	0.73 ± 0.20	0.79 ± 0.20
IPR 50 ng Cry1Ab/ml	1.02 ± 0.13	1.04 ± 0.21	0.80 ± 0.12*	1.50 ± 1.26	0.81 ± 0.21	0.82 ± 0.22
IPR 500 ng Cry1Ab/ml	1.10 ± 0.24	1.01 ± 0.24	0.98 ± 0.15	1.31 ± 0.72	0.88 ± 0.33	0.86 ± 0.23
IPR 50 ng non-GM maize protein/ml	0.93 ± 0.11	0.99 ± 0.06	0.91 ± 0.20	0.96 ± 0.10	0.87 ± 0.23	1.12 ± 0.54
IPR 500 ng non-GM maize protein/ml	0.80 ± 0.06	0.86 ± 0.14	0.77 ± 0.19	0.96 ± 0.18	0.92 ± 0.29	0.78 ± 0.18
IPR 5 µg non-GM maize protein/ml	0.77 ± 0.24	0.68 ± 0.20	0.64 ± 0.12	0.98 ± 0.27	0.86 ± 0.14	0.96 ± 0.40
IPR 50 ng GM maize protein/ml	1.00 ± 0.14	0.90 ± 0.09	0.82 ± 0.13	0.88 ± 0.14	0.83 ± 0.23	0.86 ± 0.10
IPR 500 ng GM maize protein/ml	0.85 ± 0.09	0.92 ± 0.11	0.76 ± 0.14	0.99 ± 0.23	0.84 ± 0.17	0.82 ± 0.20
IPR 5 µg GM maize protein/ml	0.97 ± 0.17	0.70 ± 0.16*	0.75 ± 0.09	0.83 ± 0.22	0.83 ± 0.26	0.93 ± 0.26

Spleen cells were incubated for 3 days with concanavalin A, phytohemagglutinin or pokeweed mitogen and for 6 days with Cry1Ab, the near isogenic non-GM maize protein extract or the GM maize protein extract in the given amounts. The table lists the indexed proliferative response (IPR) = proliferative response of stimulated cells/proliferative response of non-stimulated cells, expressed as cage mean ± SD (five cages; *n* = 10 rats)

*Statistically significant difference to the control value based on the 95% confidence interval of the SES

The cytokine production by spleen cells from male and female rats of the feeding trials D and E incubated with concanavalin A or Cry1Ab is shown in Tables 12 and 13, respectively. IL-2, IL-4, IL-10, IL-17A and TNF-α were detected in the supernatant of spleen cells incubated with concanavalin A and IL-10 was present in the supernatant of spleen cells incubated with Cry1Ab, whereby their levels did not significantly differ between the experimental groups in both feeding trials. IL-1α, IL-1β, IL-5, IL-6, IL-12p70, IL-13, G-CSF, GM-CSF and TNF-α were below the detection limit of the corresponding assays. The IFN-γ assay did not deliver biologically consistent results in a first step and could not be repeated, since no samples were available anymore.

**Table 12:** Cytokine production by spleen cells from male and female rats of feeding trial D incubated with concanavalin A or Cry1Ab

Parameter	Male rats			Female rats		
	Control	11% GMO	33% GMO	Control	11% GMO	33% GMO
Interleukin-2 (pg/ml; Con A)	6339 ± 1364^a^	5759 ± 1921^b^	6635 ± 1015	5549 ± 756	5743 ± 1130	5399 ± 871
Interleukin-4 (pg/ml; Con A)	11.99 ± 4.78	12.13 ± 3.99	12.82 ± 5.15	36.86 ± 59.32	19.62 ± 15.78	18.69 ± 12.20
Interleukin-10 (pg/ml; Con A)	4335 ± 2855	4650 ± 4758	3901 ± 2477	2271 ± 1076	2896 ± 2111	2246 ± 1379
Interleukin-17A (pg/ml; Con A)	224 ± 85	304 ± 236	313 ± 179	265 ± 58	421 ± 328	257 ± 120
Tumour necrosis factor-α (pg/ml Con A)	63.97 ± 15.34	56.20 ± 19.12^a^	53.02 ± 7.50	48.76 ± 7.83	53.81 ± 9.33	44.76 ± 11.42
Interleukin-10 (pg/ml; Cry1Ab)	413 ± 165	219 ± 64	388 ± 130	253 ± 70	308 ± 89^a^	269 ± 143

Spleen cells were incubated for 3 days with concanavalin A (Con A) or for 6 days with Cry1Ab. The table lists the amount of cytokines released into the cell culture medium, expressed as cage mean ± SD (five cages; *n* = 10 rats, if not otherwise stated)

^a^*n* = 9

^b^*n* = 8

**Table 13:** Cytokine production by spleen cells from male and female rats of feeding trial E incubated with concanavalin A or Cry1Ab

Parameter	Male rats			Female rats		
	Control	11% GMO	33% GMO	Control	11% GMO	33% GMO
Interleukin-2 (pg/ml; Con A)	7022 ± 1105^c^	6291 ± 1373^b^	6786 ± 1327^c^	5096 ± 982	5809 ± 933	6456 ± 861^a^
Interleukin-4 (pg/ml; Con A)	12.70 ± 4.94	9.64 ± 4.35	7.55 ± 1.06	12.95 ± 5.10	17.31 ± 10.32	42.90 ± 40.33
Interleukin-10 (pg/ml; Con A)	3787 ± 2771	2873 ± 1603	3273 ± 1692	2067 ± 1670	3666 ± 3053	2204 ± 1461
Interleukin-17A (pg/ml; Con A)	268 ± 157	239 ± 96	255 ± 148	311 ± 241	321 ± 159	370 ± 148
Tumour necrosis factor-α (pg/ml Con A)	62.23 ± 14.10	52.51 ± 12.51	69.66 ± 20.32	43.51 ± 10.38	47.69 ± 8.36^a^	57.14 ± 16.68
Interleukin-10 (pg/ml; Cry1Ab)	310 ± 45	278 ± 67	392 ± 129	360 ± 187^a^	329 ± 69	537 ± 159

Spleen cells were incubated for 72 h with concanavalin A (Con A) or for 144 h with Cry1Ab. The table lists the amount of cytokines released into the cell culture medium, expressed as cage mean ± SD (5 cages, *n* = 10 rats, if not otherwise stated; four cages in the case of *n* = 7 rats)

^a^*n* = 9

^b^*n* = 8

^c^*n* = 7

The SES graphs with the complete set of parameters measured in the studies D and E are shown in the Supplementary Material (Figs. 2A–D, 3A–D, respectively). A summary of the statistically significant parameter differences between control and MON810-fed rats in the trials D and E is shown in Table 14.

**Table 14:** Summary of the statistically significant parameter differences between control and MON810-fed rats in the trials D and E

Parameter	Study D				Study E			
	11% GMO		33% GMO		11% GMO		33% GMO	
	Male	Female	Male	Female	Male	Female	Male	Female
Anti-maize-specific IgG level					↑			
% CD3^+^CD4^+^ cells in the thymus			↓					
% CD3^+^CD4^+^ cells in the spleen								↑
% CD3^+^ cells in the spleen								↑
% CD3^−^ cells in the spleen								↓
Phagocytic activity of granulocytes		↑						
Respiratory burst in phagocytes		↑						
Proliferative response of spleen cells to phytohemagglutinin			↓		↓			
Proliferative response of spleen cells to MON810 maize protein					↓			
Proliferative response of spleen cells to Cry1Ab							↓	

## Discussion

In the present study, the impact of feeding MON 810 maize on the immune responses of rats was assessed by measuring total and specific antibodies to Cry1Ab and maize proteins, phagocytic activity and responses to mitogenic stimulation. Regarding a potential immunogenicity of Cry1Ab in the MON810 maize, antibodies against Cry1Ab were not produced in the rats fed the MON810 maize at dietary incorporation levels of 11 or 33%. This was also the case in the preliminary experiments, in which the rats were intraperitoneally immunized with the GM maize to obtain antisera to develop and validate the immunoassays. These findings are in accordance with a study by Kroghsbo et al. (2008), in which the authors reported that Cry1Ab induced specific immune responses in Wistar rats depending on the route of exposure, i.e. Cry1Ab induced them when inhaled, but not when ingested. In line with this observation, Andreassen et al. (2015a) showed that the intranasal instillation of Cry1Ab in BALB/c mice resulted in the production of Cry 1Ab-specific IgE and IgG1 antibodies. In another study, no Cry1Ab-specific immune response was induced after the intragastric administration of Cry1Ab or the intragastric sensitization with the MON810 maize variety DKC6575 in combination with a mucosal Th2 adjuvant in BALB/c mice (Adel-Patient et al. 2011).

Regarding a potential adjuvant effect of Cry1Ab, i.e. the capacity to enhance the immunogenicity of and induce the sensitisation to an unrelated protein with which it is co-administered, Guimaraes et al. (2008) showed that the oral administration of Cry1Ab did not increase the sensitization to peanut proteins but observed a possible impact on the elicitation of the allergic reaction in the BALB/c mouse model. Adel-Patient et al. (2011) observed a significant production of IgE and IgG1 antibodies specific to maize proteins induced after intragastric sensitization with an extract of MON810 maize in the presence of a mucosal Th2 adjuvant in BALB/c mice when compared to PBS-treated mice. However, there were no differences in the IgE and IgG1 antibody responses to maize proteins between mice treated with MON 810 or the conventional maize. In the present study, a statistically significant alteration in the anti-maize protein antibody response was observed in male rats fed 11% GMO in trial E, but this increase was not observed at the 33% dietary incorporation level. Hence, the results obtained in the feeding trials D and E confirm that Cry1Ab does not exert an allergenic or an adjuvant activity at the concentrations at which it is expressed in the two tested cultivars.

A systemic and mucosal adjuvant activity was described after the intraperitoneal, intranasal and intragastric administration (in the latter case in the presence of magnesium–aluminium hydroxide) of a high dose of Cry1Ac, a *Bacillus thuringiensis* protein that is structurally and functionally similar to Cry1Ab but is not expressed in the MON810 maize variety, to laboratory animals. In particular, an increased antibody response against an unrelated protein (i.e. ovalbumin) was observed (Vázquez-Padrón et al. 1999; González-González et al. 2015; Moreno-Fierros et al. 2015). In contrast, the adjuvant effect of Cry1Ab on ovalbumin was not observed in BALB/c mice after airway exposure to extracts of MON810 pollen/leaf or trypsinized Cry1Ab (Andreassen et al. 2015b). Thus, the issue of adjuvanticity seems to be related to the exposure conditions and, particularly, to the administered doses, although very little is known regarding the dose–response relationship to induce this effect.

In trial E, the increase in the percentage of CD3^+^ cells and CD3^+^CD4^+^ cells as well as the decrease in the percentage of CD3^−^ cells in the spleen were restricted to female rats fed the 33% GMO diet, but not observed in any other experimental group, and are not indicative of any pathophysiological process, i.e. are of no toxicological relevance. In this context, it should be noted that no histopathological changes were observed in the spleens of the male and female rats fed the 11% GMO and 33% GMO diets in Trials D and E (Schmidt et al. 2017). The possibility that the MON810 maize could affect the phagocytic activity of granulocytes and/or with the respiratory burst of phagocytes after incubation with *Staphylococcus aureus* bacteria was also analyzed. Only the percentage of phagocytic granulocytes and the percentage of phagocytes showing a respiratory burst were statistically higher in female rats fed the 11% GMO diet than in the control group in study D, but these alterations were not observed when female rats were fed the 33% GMO diet and are thus considered of no toxicological significance. Moreover, to determine whether the MON810 maize could interfere with the ability of spleen cells to undergo a clonal proliferation when stimulated in vitro with concanavalin A, phytohemagglutinin, pokeweed mitogen, Cry1Ab, a near isogenic non-GM maize protein extract or a GM maize protein extract, lymphocyte proliferation assays were performed. It has to be noted that alterations in the proliferative response of spleen cells were sporadically observed and, if so, were either not reproduced in both trials, were only observed with one out of five stimuli and/or did not occur concentration-dependently, so that they are considered to be of no toxicological relevance. The only cytokine to be decreased after an incubation of spleen cells from male and female rats with Cry1Ab was interleukin-2 in female rats fed the 11% GMO diet in Trial E. This alteration was not observed in female rats fed the 33% GMO diet in Trial E and not observed at all in the Trial D, so that it is considered to be of no toxicological significance.

Taken together, only single parameters were sporadically altered in rats fed the MON810 maize when compared to control rats, and these alterations are considered to be of no immunotoxicological significance.

However, a long-term and continuous airway exposure to small amounts of the Cry1Ab protein could occur in workers handling genetically modified plants expressing this protein in farms and factories. Information regarding the immunological effects of long-term airway exposure to Cry1Ab is limited. Therefore, it would be advisable to investigate immune responses in workers at a risk of airway exposure to Cry1Ab protein.

Our data are in accordance with the conclusions and recommendations provided by the GRACE project (http://www.grace-fp7.eu), i.e. there is no indication that the performance of 90-day feeding studies with whole food/feed would provide additional information on the safety of the GM maize MON810 if compared to the compositional analysis of the GM line and its conventional counterpart (i.e. the genetically closest non-GM comparator) in terms of an initial safety assessment.

In line with the GRACE transparency policy, any interested person will have access to the raw data of studies D and E obtained in the frame of the GRACE project through an internet portal named CADIMA (*C*entral *A*ccess *D*atabase for *Im*pact *A*ssessment of Crop Genetic Improvement Technologies; http://www.cadima.info).

## Electronic supplementary material

Below is the link to the electronic supplementary material.

Supplementary material **Fig. 1**. (A) IgE standard curve. (B) Assessment of the intra/inter-assay variability by reproducing the IgE immunoassay on various plates on the same day or on two separate days (1 week apart) with the selected IgE antibody (CV, coefficient of variation). mAbs414nm, absorbance unit at 414 nm. (PPTX 246 KB)

Supplementary material **Fig. 2**. Standardized effect size graphs for the comparison of the humoral and cellular immune response between control and 11% GMO-fed female rats (A), between control and 33% GMO-fed female rats (B), between control and 11% GMO-fed male rats (C) and between control and 33% GMO-fed male rats (D) in the 90-day feeding trial D. (PDF 246 KB)

Supplementary material 3 (PDF 246 KB)

Supplementary material 4 (PDF 246 KB)

Supplementary material 5 (PDF 246 KB)

Supplementary material **Fig. 3**. Standardized effect size graphs for the comparison of the humoral and cellular immune response between control and 11% GMO-fed female rats (A), between control and 33% GMO-fed female rats (B), between control and 11% GMO-fed male rats (C) and between control and 33% GMO-fed male rats (D) in the 90-day feeding trial E. (PDF 246 KB)

Supplementary material 7 (PDF 246 KB)

Supplementary material 8 (PDF 245 KB)

Supplementary material 9 (PDF 245 KB)
