# Supplementary figures and images for: Humoral and cellular immune response in Wistar Han RCC rats fed two genetically modified maize MON810 varieties for 90 days (EU 7th Framework Programme project GRACE)
Source: Arch Toxicol. 2018 May 31;92(7):2385–99. doi: 10.1007/s00204-018-2230-z (PMC6015625; doi:10.1007/s00204-018-2230-z)

## Slide 1
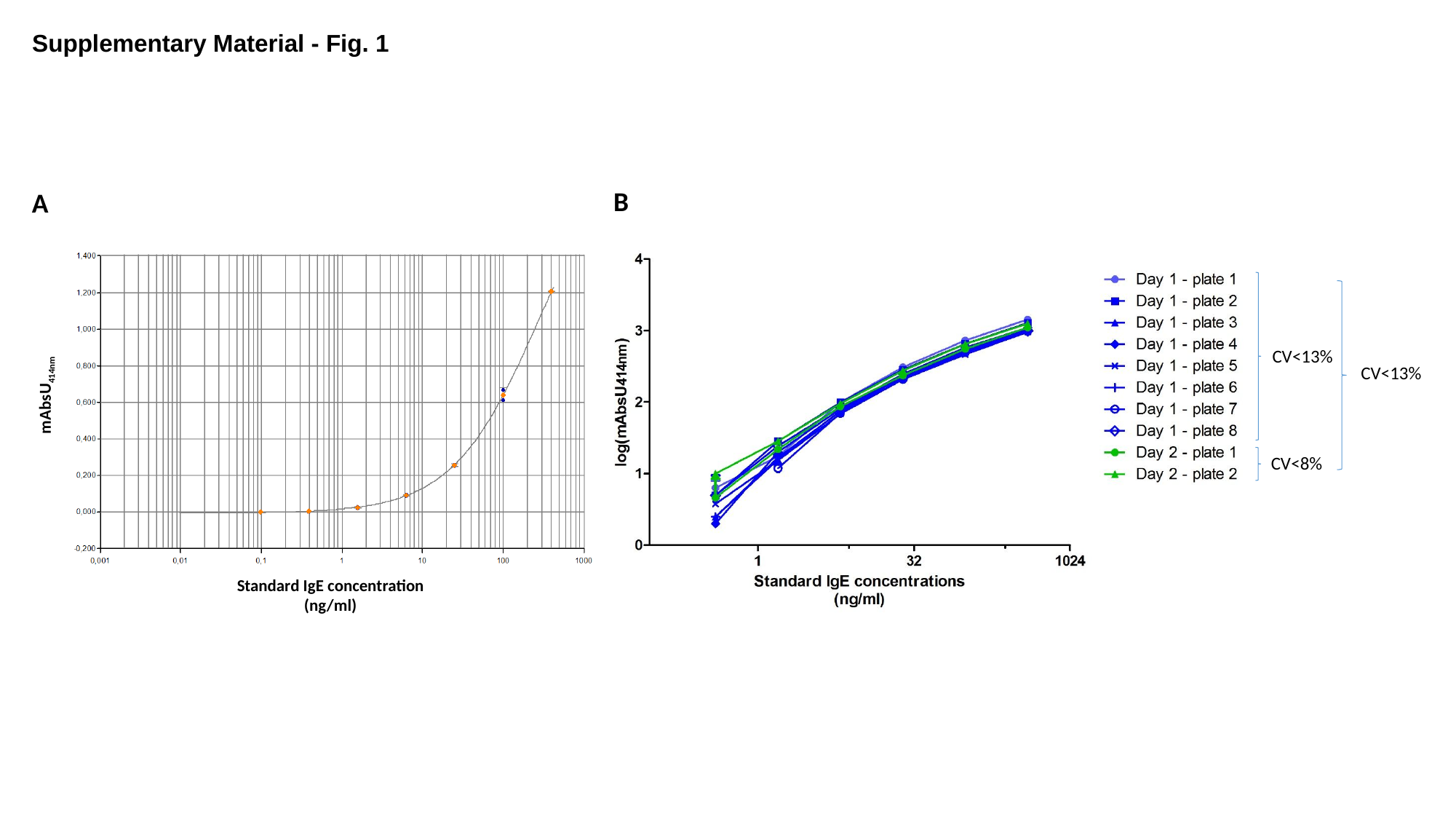

Supplementary Material - Fig. 1
B
A
mAbsU414nm
Standard IgE concentration
(ng/ml)
CV<13%
CV<13%
CV<8%

Supplement: Supplementary file 1 — Supplementary material Fig. 1. (A) IgE standard curve. (B) Assessment of the intra/inter-assay variability by reproducing the IgE immunoassay on various plates on the same day or on two separate days (1 week apart) with the selected IgE antibody (CV, coefficient of variation). mAbs414nm, absorbance unit at 414 nm. (PPTX 246 KB) [file 204_2018_2230_MOESM1_ESM.pptx]

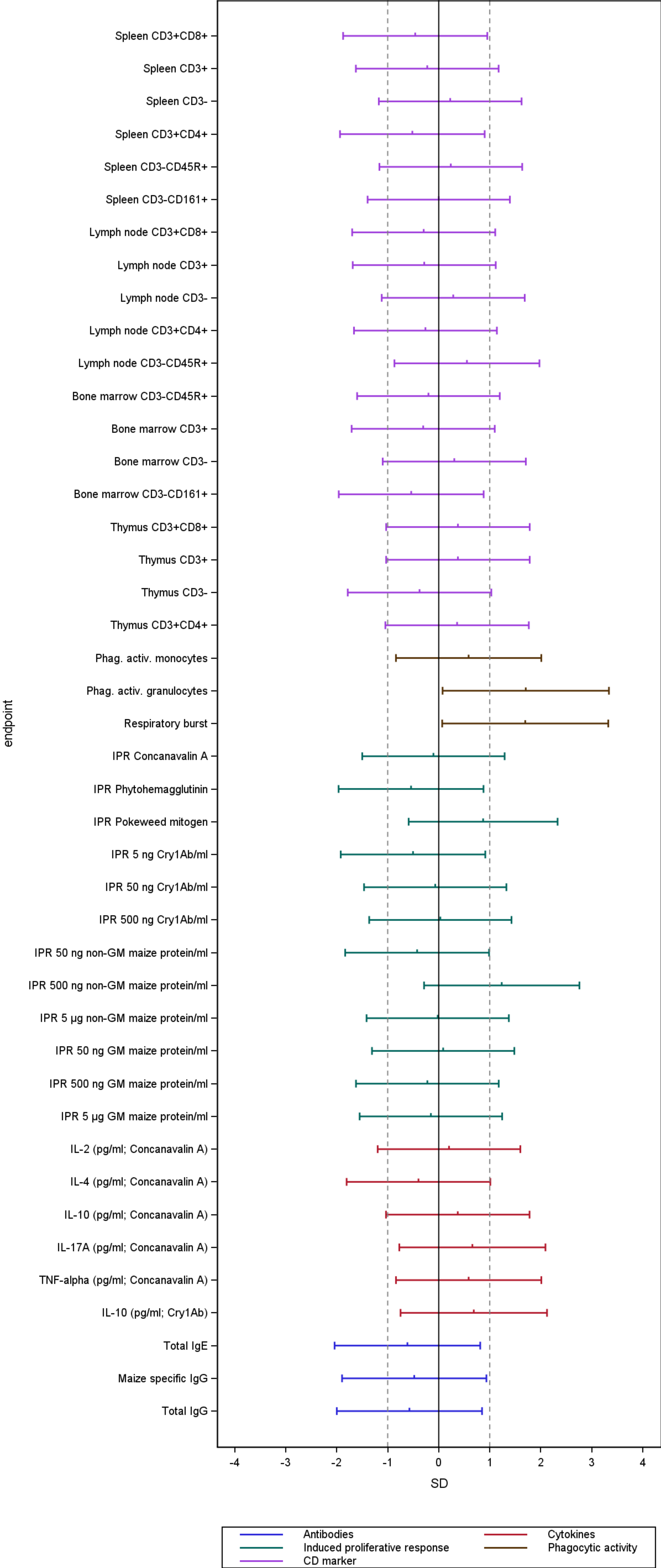

Supplement: Supplementary file 2 — Supplementary material Fig. 2. Standardized effect size graphs for the comparison of the humoral and cellular immune response between control and 11% GMO-fed female rats (A), between control and 33% GMO-fed female rats (B), between control and 11% GMO-fed male rats (C) and between control and 33% GMO-fed male rats (D) in the 90-day feeding trial D. (PDF 246 KB) [file 204_2018_2230_MOESM2_ESM.pdf]

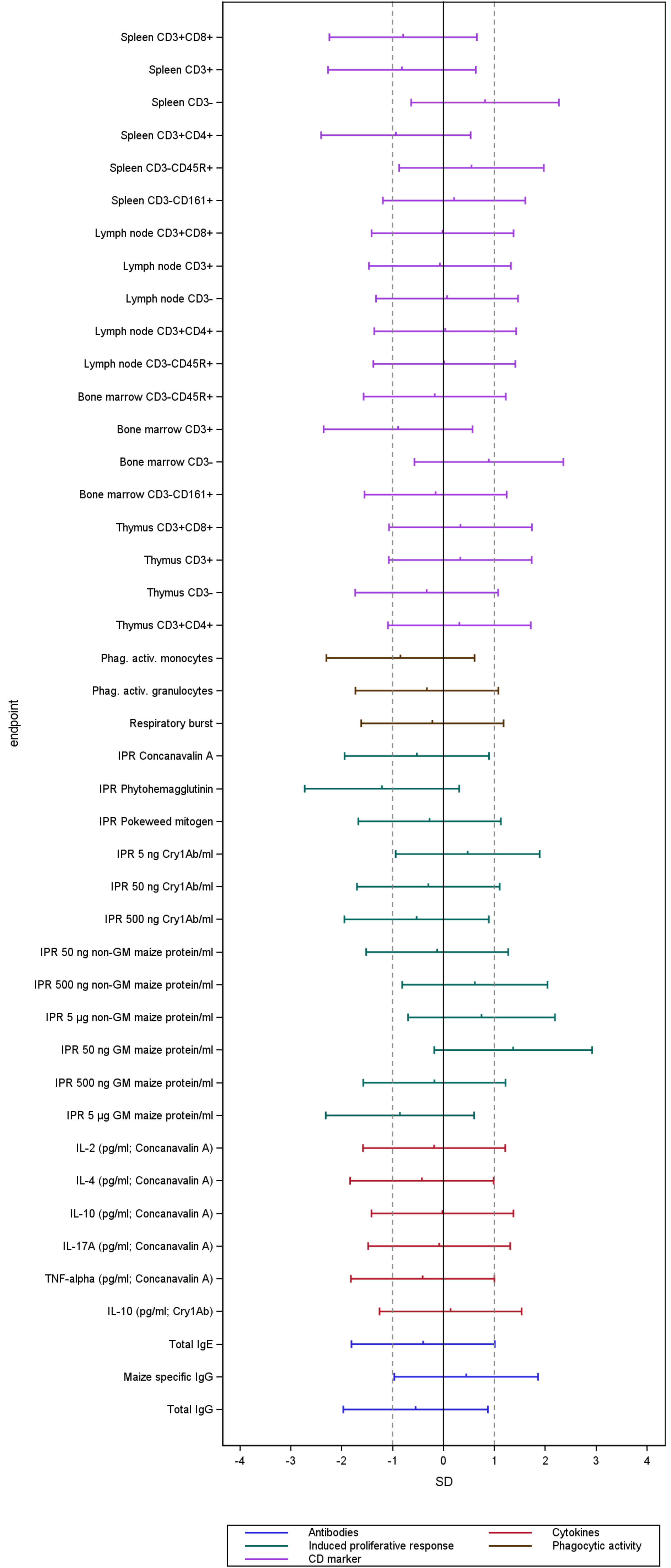

Supplement: Supplementary file 3 — Supplementary material 3 (PDF 246 KB) [file 204_2018_2230_MOESM3_ESM.pdf]

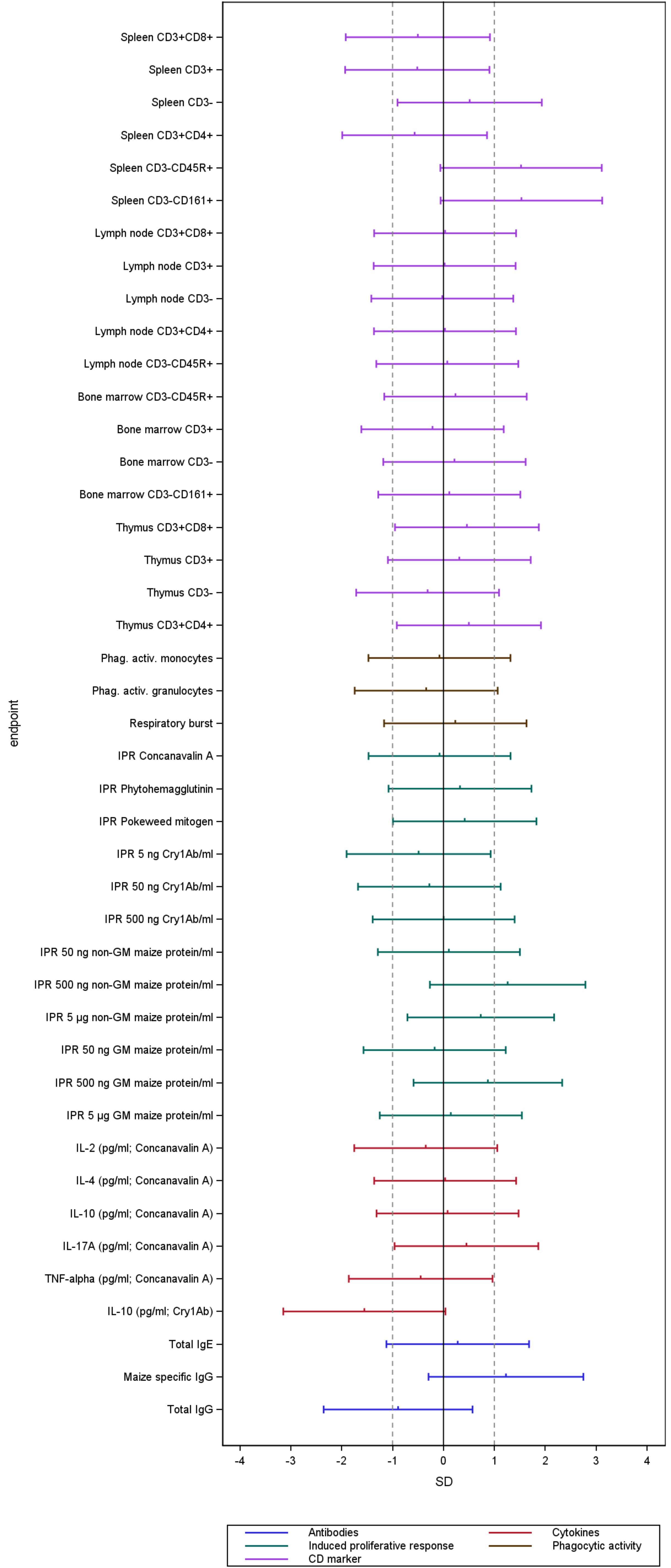

Supplement: Supplementary file 4 — Supplementary material 4 (PDF 246 KB) [file 204_2018_2230_MOESM4_ESM.pdf]

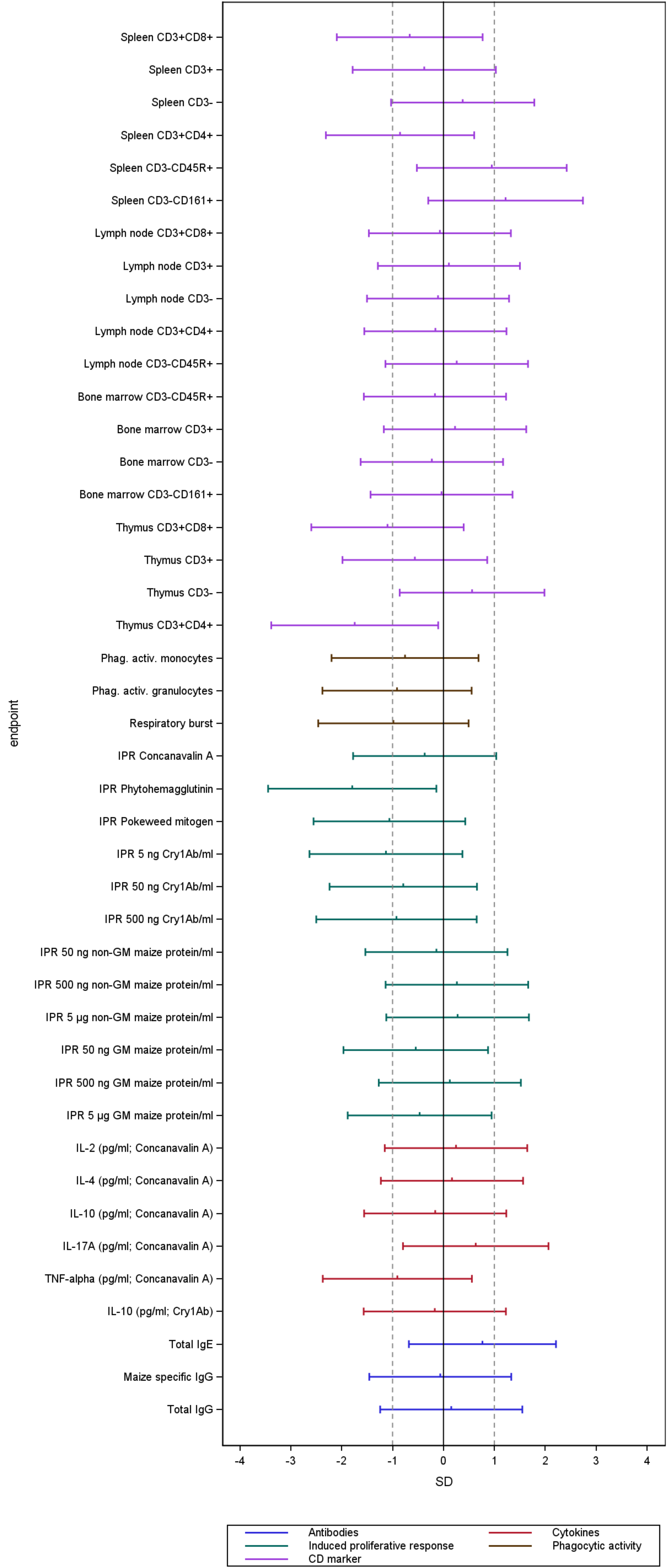

Supplement: Supplementary file 5 — Supplementary material 5 (PDF 246 KB) [file 204_2018_2230_MOESM5_ESM.pdf]

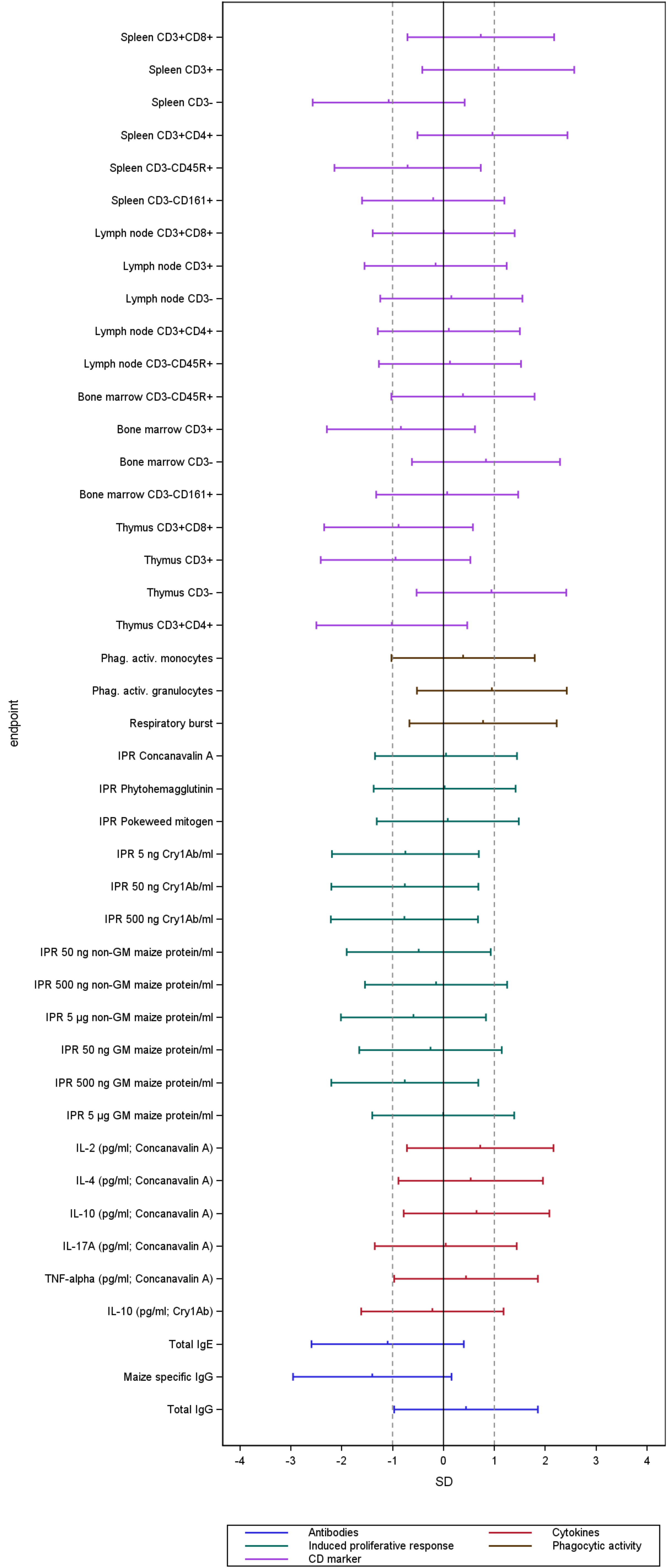

Supplement: Supplementary file 6 — Supplementary material Fig. 3. Standardized effect size graphs for the comparison of the humoral and cellular immune response between control and 11% GMO-fed female rats (A), between control and 33% GMO-fed female rats (B), between control and 11% GMO-fed male rats (C) and between control and 33% GMO-fed male rats (D) in the 90-day feeding trial E. (PDF 246 KB) [file 204_2018_2230_MOESM6_ESM.pdf]

sex=female comparison=33% GMO - control

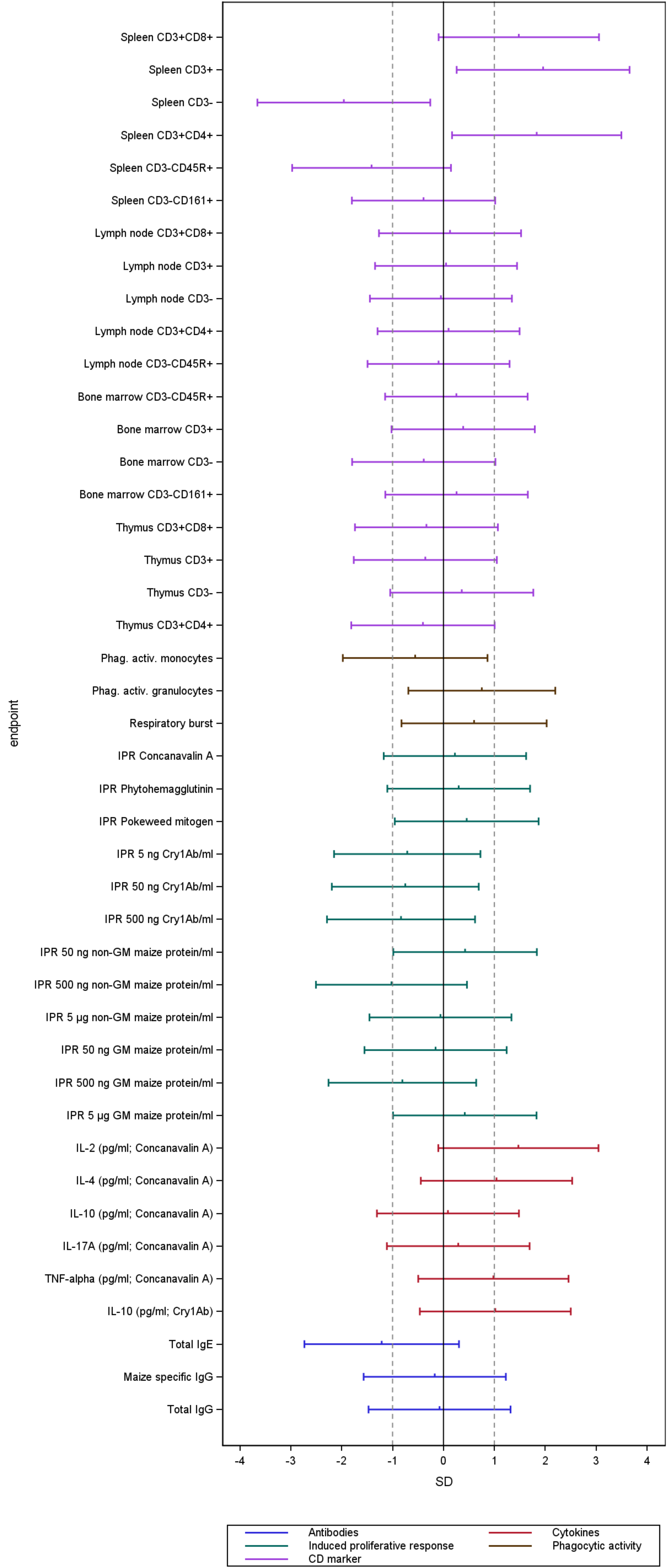

Supplement: Supplementary file 7 — Supplementary material 7 (PDF 246 KB) [file 204_2018_2230_MOESM7_ESM.pdf]

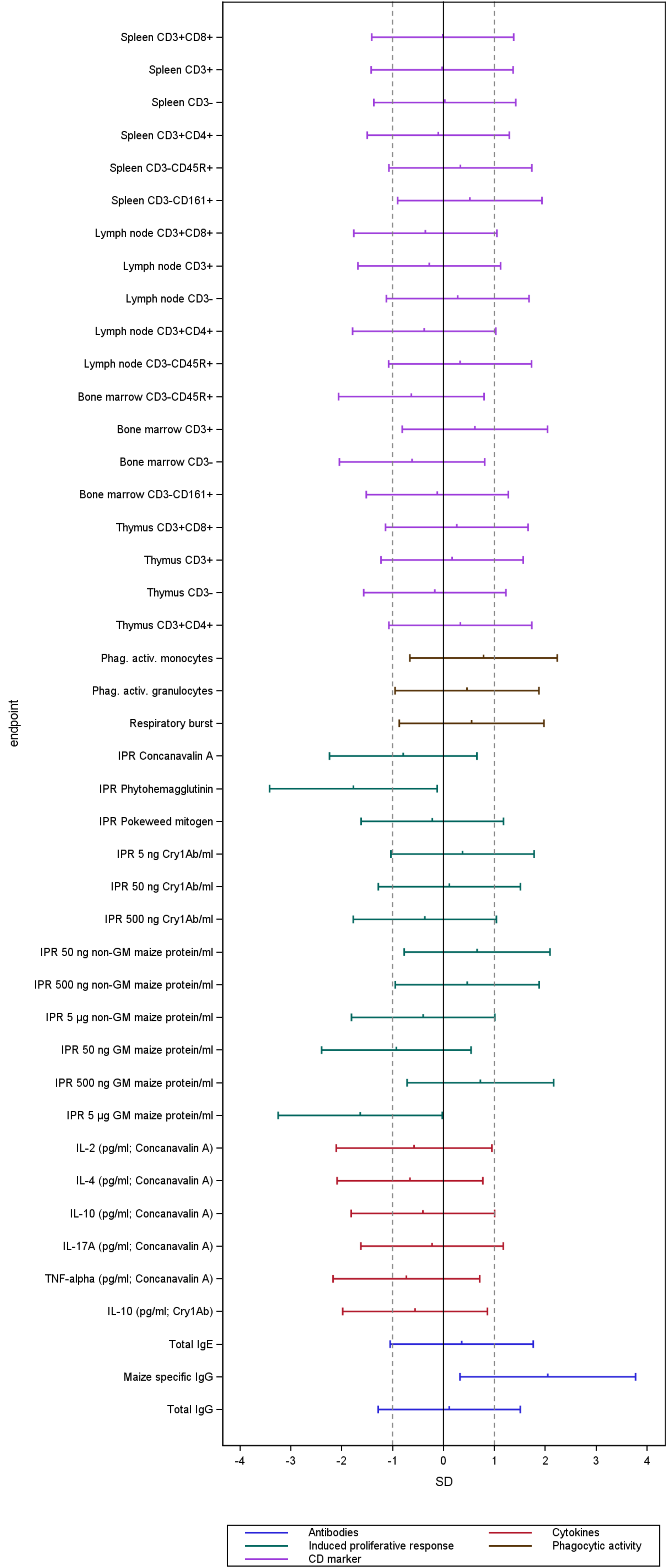

Supplement: Supplementary file 8 — Supplementary material 8 (PDF 245 KB) [file 204_2018_2230_MOESM8_ESM.pdf]

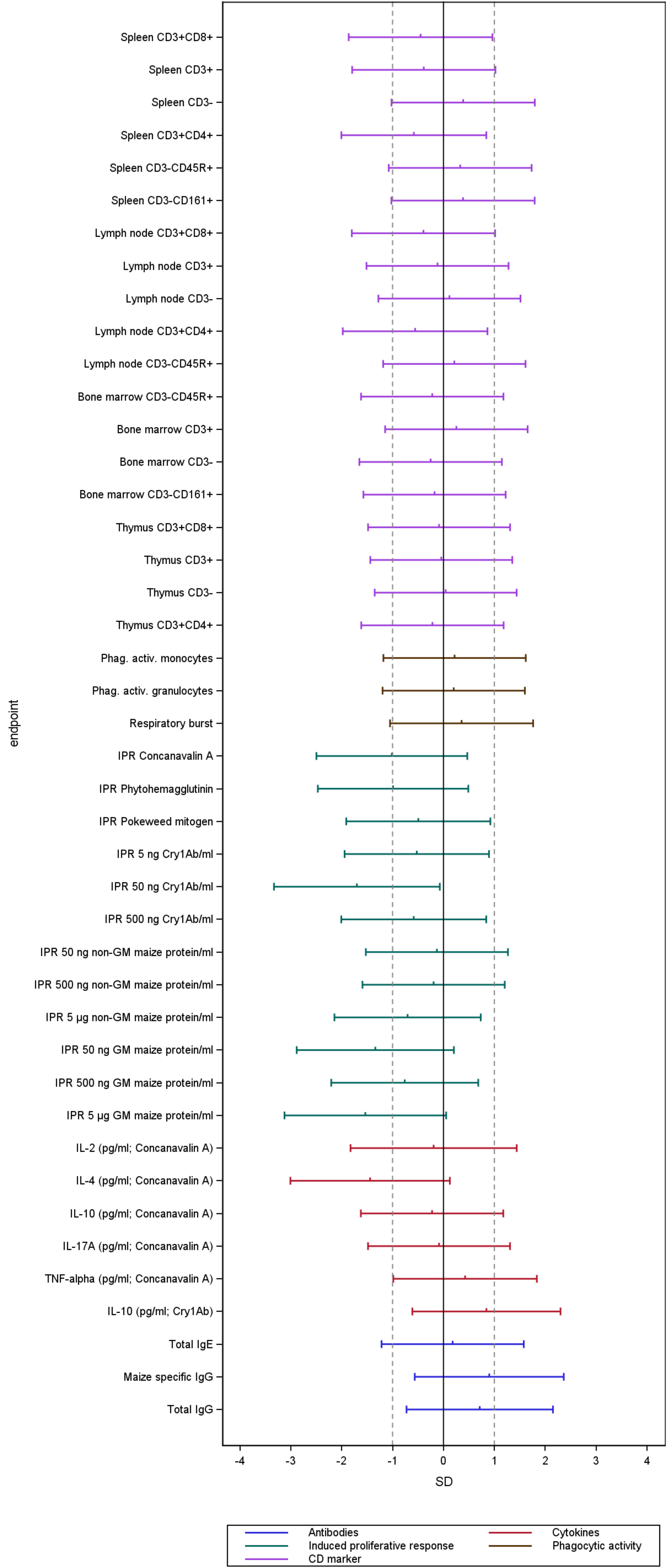

Supplement: Supplementary file 9 — Supplementary material 9 (PDF 245 KB) [file 204_2018_2230_MOESM9_ESM.pdf]
